# The Effect of Case-Based Instruction on Physician Assistant Pharmacology Education

**DOI:** 10.7759/cureus.106770

**Published:** 2026-04-10

**Authors:** Laney Henley, Seth M Alexander, Nicole Mihailovich, Jeanette Elfering, James J Fiordalisi

**Affiliations:** 1 Pharmacology, University of North Carolina School of Medicine, Chapel Hill, USA; 2 Health Sciences, University of North Carolina School of Medicine, Chapel Hill, USA; 3 Internal Medicine, Vanderbilt University Medical Center, Nashville, USA; 4 Pediatrics, Vanderbilt University Medical Center, Nashville, USA

**Keywords:** case-based learning, comparative teaching, learning retention, pharmacology, physician assistant education

## Abstract

Background: Case-based learning (CBL) is a widely adopted instructional method in medical education, guiding students through real-world scenarios to accomplish specific learning objectives. Despite literature supporting its use in medical education at the Undergraduate Medical Education (UME) level and in some other health professions, there is a lack of research surrounding its utility in physician assistant (PA) education compared to the more traditional, lecture-based instruction. This difference is particularly true in pharmacology, a subject well-suited for the application of basic science to clinical medicine through CBL.

Methods: Fifteen first-year PA students enrolled in an entry-level pharmacology course participated in six pharmacology learning cycles, each learning cycle consisting of a discrete didactic session followed by a corresponding case-based learning session on the same topic. All first-year students enrolled in the program were allowed to participate voluntarily in the data collection; however, the curriculum was required for all students. For each topic, students completed multiple-choice evaluations at three time points: before didactic teaching sessions, after didactic teaching sessions but before case-based sessions, and after case-based sessions. At the conclusion of the six learning cycles, students completed a 12-item Likert survey to assess their perspectives on the CBL sessions.

Results: The average student score across all six case evaluations demonstrated a statistically significant increase in average scores from predidactic (mean = 31% correct; SD = 8.0) to the postdidactic evaluations (p = 0.01; mean = 42%; SD = 13), and then again to postdidactic to post-CBL evaluations (p < 0.01; mean = 53%; SD = 15). Evaluations within individual learning cycles, however, showed varying results across assessments. Students felt CBL improved their understanding and prepared them for their board exams (73.33% agree or strongly agree, N = 11). However, students had slightly mixed opinions about how useful CBL was for their understanding of the learning topics compared with their didactics, and positive opinions on CBL being introduced as a new learning method and its impact on board exam preparation.

Conclusions: CBL is an effective learning method in supplementing traditional didactic teaching in PA pharmacology education, leading to significant learning gains and favorable student feedback. Unlike prior studies that compared CBL directly with conventional teaching methods, this study demonstrates the supplementary benefit of CBL.

## Introduction

Thistlethwaite et al. define case-based learning (CBL) sessions as “a learning and teaching approach that … link theory to practice, through the application of knowledge to the cases, and encourage the use of inquiry-based learning methods.” Since its first description in the literature in 1912, CBL has grown in popularity as a useful and interactive teaching method, particularly in medical education [1]. A meta-analysis of eight randomized-controlled trials comparing CBL with traditional didactic teaching in medical education found CBL to be an effective method for improving academic performance and ability in measures of skill regarding case analysis [2]. Additionally, for medical students, student perceptions of educational quality are higher for case-based teaching methods [3].

While an effective instructional strategy for medical students [4], there remains limited research on the application of CBL teaching to physician assistant (PA) students. Literature within PA education analyzing the effects of problem-based learning (PBL), a similar style of active learning engaging students with real-life scenarios, has been published [5]. While both are built on principles of adult learning theory, PBL differs from CBL in that PBL focuses primarily on creating solutions to a realistic problem. In contrast, CBL focuses on the application of knowledge to multiple aspects of a real-world case and context. In at least one study examining outcomes of PBL, there was no difference in the Physician Assistant National Certifying Exam (PANCE) scores when compared to didactic teaching [6]. PBL learning methods within PA education were shown to increase learner-reported confidence in understanding of the course material, however [7].

Much of the body of research into the utility of CBL exists in direct comparison of CBL to didactic teaching within medical student education. This research has shown similar knowledge-based learning outcomes but improved learning and skill acquisition within communication and team-based problem-solving domains [2,4,8]. CBL used in combination with didactic traditional lecture-based teaching is an area that, despite being deployed in practice, has not been described in the literature as thoroughly.

Pharmacology as a discipline lends itself to case-based instruction, as the application of basic sciences to clinical practice is a critical skill for safely and effectively prescribing medications. In pharmacology specifically, CBL helps develop higher-order skills of analysis and application [9] as well as increasing scores on higher-level Bloom’s taxonomy questions [10] for medical students. However, with both objective and subjective outcomes supporting CBL in medical student education and support for this pedagogy in pharmacology education, there is limited research on its use in PA education. 

Given these gaps in the literature, this study seeks to investigate the effect of CBL in PA pharmacology education as a supplement to didactic teaching through the analysis of objective knowledge-based assessments and subjective student evaluations of the curriculum. 

## Materials and methods

Curriculum design 

First-year PA students at the University of North Carolina at Chapel Hill PA program participate in three semester-long basic pharmacology courses focusing on foundational principles and their relation to clinical applications. Topics in the first course’s curriculum include the treatment of hypertension, angina, atrial fibrillation, hyperlipidemia, asthma, and pneumonia. Each of these topics was first presented in a didactic, lecture-based instructional session. An interdisciplinary team of basic science and clinical PA faculty created a clinically-oriented case study to follow the initial didactic session. These cases consist of a theoretical patient’s history of present illness, medical history, social history, vitals, and physical exam findings and were reviewed in a separate session. Case-based sessions required students, in groups of five, to review and discuss the case and complete associated questions that elaborated on the clinical applications of foundational pharmacology concepts. The course director, who has a significant background in pharmacology education, served as the sole facilitator for all case discussions. At the end of each case session, an in-depth discussion of the key foundational and clinical points was mediated by the course instructor. For each of the learning cycles, a multiple-choice assessment (herein "assessment(s)") was created that focused on concepts taught in both the didactic and CBL sessions. 

Learning cycles and assessments 

The study was composed of six learning cycles during a four-month instructional period. Each learning cycle was associated with one of the topic areas described previously. Six accompanying clinical vignette-based, multiple-choice assessments were created by an interdisciplinary faculty team based on the learning objectives for each content area/learning cycle; there were an average of 9.5 questions per assessment, reviewed by all faculty. Each learning cycle consisted of five parts: a baseline assessment prior to instruction, then a didactic-based teaching session, followed by a postdidactic/precase administration of the same multiple-choice assessment. Next, students participated in a CBL session and subsequently completed the same assessment one final time. Each distinct, didactic, or case-based teaching session occurred in person over a 90-minute class session. Each assessment (predidactic, postdidactic, and postcase) was administered within a week of the respective session. To limit recall bias due to the use of the same multiple-choice assessment, they were administered through the institutional learning management system (Canvas LMS, Instructure Inc., Salt Lake City, UT), where scores and questions were only released to students at the conclusion of the learning cycle. Additionally, grading of the multiple-choice assessments was based on completion, not performance. This study design was chosen to prevent additional bias introduced by using different assessments of potentially varying difficulty, limiting comparative utility. Comparative statistics were completed using STATA Basic Edition (BE) Version 17.0 (Released 2021; StataCorp LLC, College Station, TX). Given the sample size and that several subgroups did not meet the parametric assumption of normality, Wilcoxon signed-rank testing was utilized in lieu of parametric analysis. 

Survey structure 

A confidential, Likert-scale survey was designed to evaluate student perceptions of both case-based and didactic teaching from the previous semester within their pharmacology coursework. The survey questions were adopted from an open-access survey [11] and modified minimally by the research team from its previous use among paramedic students to PA students for better application to our study population. Modifications were reviewed by a separate member of the faculty (Alexander) to determine if the integrity of the instrument was maintained. The student feedback questionnaire consisted of 12 questions on a five-point Likert-type scale (1 = strongly disagree; 2 = disagree; 3 = neither agree nor disagree; 4 = agree; 5 = strongly agree). Students were surveyed via a confidential Qualtrics survey (Qualtrics XM, Provo, UT) distributed to each student via their institutional email at the conclusion of all six learning cycles. Summative, descriptive statistics for the survey results are reported directly from the survey software. This study was reviewed by the Institutional Review Board of the University of North Carolina at Chapel Hill, which determined it to be exempt from human subject research regulations (IRB Study 25-0263). 

## Results

**Figure 1:**
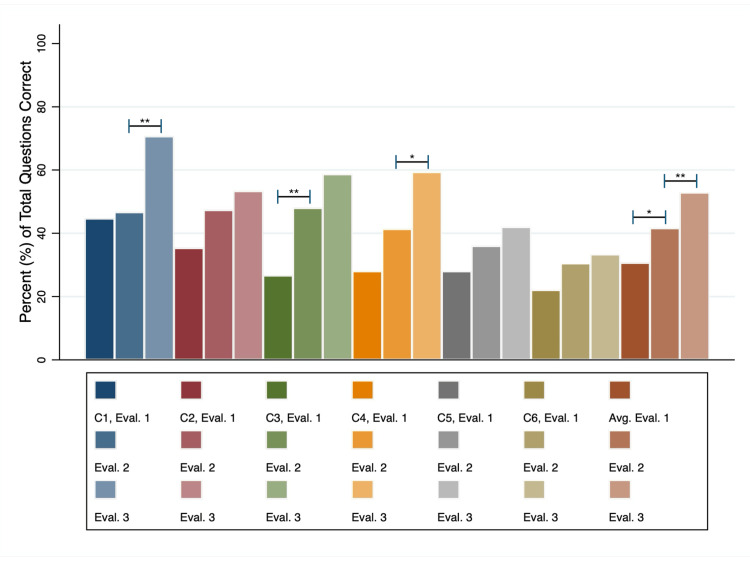
The average score for each learning assessment (Eval.) as a percentage of total questions per assessment Each learning cycle (C) is identified by bars of three associated colors representing the three assessments. Asterisks indicate the degree of significant differences between average scores of any two sequential assessments (p = 0.01-0.05 (*) or p < 0.01 (**))

Fifteen students responded to the distributed survey and consented to participate in the study out of the twenty eligible students enrolled in the curriculum (N = 15; 75% response rate). Cumulative results from all six learning cycles indicate significant increases in average scores from predidactic (assessment 1, mean = 31% correct; SD = 8.0) to postdidactic assessment (assessment 2, mean = 42%; SD = 13; p = 0.01) and from postdidactic to post-CBL assessments (assessment 3, mean = 53%; SD = 15; p < 0.01). Each learning cycle showed score increases across assessment timepoints, some with statistically significant gains and others without statistical significance (Figure 1). Assessments are compared using the Wilcoxon signed-rank tests in Table 1. 

**Table 1:** Comparative statistical testing between the first, second, and third assessment scores (expressed as a percentage of points available) throughout the didactic and case-based pharmacology curriculum Wilcoxon signed-rank testing was utilized given the sample size (test statistic = z-score). Statistical significance is denoted within the table (*)

Learning cycle	Assessment 1 (mean, SD)	Assessment 2 (mean, SD)	Assessment 3 (mean, SD)	Assessment 1 to assessment 2 (test statistic, p-value)	Assessment 2 to assessment 3 (test statistic, p-value)
First	45, 20	47, 24	71, 21	0.37, 0.71	3.40, <0.01 *
Second	35, 18	47, 22	53, 39	1.49, 0.14	1.18, 0.27
Third	27, 14	48, 21	59, 23	2.98, <0.01 *	1.55, 0.14
Fourth	28, 15	41, 23	59, 23	1.90, 0.06	2.29, 0.02*
Fifth	28, 15	36, 17	42, 20	1.77, 0.08	0.72, 0.49
Sixth	22, 9.4	30, 19	33, 23	0.70, 0.49	0.76, 0.45
Overall average	31, 8.0	42, 13	53, 15	2.50, 0.01 *	3.04, <0.01 *

The Likert scale survey data show that 100% of respondents (N = 15) agreed that “Didactic lectures were useful in the understanding of the topic.” The majority of respondents agreed that “CBL was useful in the understanding of the topic” (86.67%, N = 13). Additionally, most respondents agreed that “CBL improved my ability to apply concepts of basic sciences to clinical situations” (73.33%, N = 11). A large portion of respondents agree with the statement “[t]raining in CBL sessions will help me in preparing for my board examinations” (73.33%, N = 11). A summary of the Likert scale responses is shown in Table 2. 

**Table 2:** Descriptive data from a survey of student perceptions of CBL and didactic teaching methods in pharmacology curriculum delivery via Likert scale CBL: case-based learning The survey was adapted from Williams et al. [11] published Open Access under a Creative Commons License (https://creativecommons.org/licenses/by-nc-nd/4.0/) with permission from the original author to publish with minor revisions as described in the methods

Statement	Strongly disagree % (N)	Disagree % (N)	Neither agree nor disagree % (N)	Agree % (N)	Strongly agree % (N)
CBL was useful in the understanding of the topic	0 (0)	6.67 (1)	6.67 (1)	80 (12)	6.67 (1)
Didactic lectures were useful in the understanding of the topic	0 (0)	0 (0)	0 (0)	26.67 (4)	73.33 (11)
The problems given in the CBL were in the context of the topic under study	0 (0)	0 (0)	6.67 (1)	53.33 (8)	40 (6)
By virtue of CBL, the drugs could be better related to their basic mechanisms	0 (0)	0 (0)	33.33 (5)	46.67 (7)	20 (3)
CBL improved my ability to apply concepts of basic sciences to clinical situations	0 (0)	13.33 (2)	13.33 (2)	60 (9)	13.33 (2)
CBL helped me develop skills in identifying potential drug-related difficulties of the patient	0 (0)	6.67 (1)	20 (3)	53.33 (8)	20 (3)
CBL improved my learning skills better than didactic lectures	13.33 (2)	26.67 (4)	33.33 (5)	26.67 (4)	0 (0)
CBL made me motivated to learn more about the topic	6.67 (1)	13.33 (2)	20 (3)	53.33 (8)	6.67 (1)
There should be a judicious mixture of didactic lectures and CBL sessions for a better understanding of drugs	0 (0)	6.67 (1)	13.33 (2)	73.33 (11)	6.67 (1)
All CBL sessions should be preceded by didactic lectures to help with better understanding	0 (0)	0 (0)	13.33 (2)	53.33 (8)	33.33 (5)
Training in CBL sessions will help me in preparing for my board examinations	6.67 (1)	6.67 (1)	13.33 (2)	66.67 (10)	6.67 (1)
CBL can be introduced as a new teaching/learning method for future classes	0 (0)	13.33 (2)	6.67 (1)	66.67 (10)	13.33 (2)

## Discussion

While not consistently statistically significant, participants' mean assessment scores increased from both predidactic to postdidactic (assessments 1 to 2) and postdidactic to postcase (assessments 2 to 3) over all six learning cycles combined. The inconsistency in which the teaching method was associated with a statistically significant increase may reflect the fact that the multiple-choice questions were created from learning objectives made to reflect didactic content. Although the content for the case-based sessions was based upon the same learning objectives created from didactic teaching, it is possible that the didactic teaching session content was more closely tested through the multiple-choice assessments. When comparing didactic sessions to CBL sessions from the respondent survey data, all respondents agreed that the didactic sessions were useful in understanding the topics, while approximately 87% (N = 13) agreed that the CBL sessions were useful. However, a portion of participants agreed that the CBL sessions improved their learning skills better than did the didactic sessions (26.67%, N = 4), and the majority of students agreed that there should be a mix of CBL and didactic-based lectures (80%, N = 12). These results suggest that students prefer a combination of both didactic teaching and CBL and that students perceive that CBL alone is less useful than didactic instruction alone. They also suggest that students prefer CBL to follow the didactic teaching, presumably because teaching foundational principles prior to clinical applications is a natural progression. Overall, a majority of students provided positive feedback for CBL in combination with traditional didactic teaching methods. This is consistent with previous studies, which have shown that when students have more time for peer-to-peer learning, their objective learning outcomes are the same, even though their perceived learning is decreased [12]. Findings such as these should be taken into account in the curricular design process for similar programs to include supplemental learning through case-based study. 

Limitations in this study include a potential bias introduced by assessment repetition, limited sample size, and the effect of outside resources or social pressures on student performance. To eliminate discrepancies in question difficulty at assessment points and decrease bias, assessment questions were identical at each assessment time point. However, this repetition could have affected objective results, as multiple-choice testing, even without feedback, stabilizes access to marginalized knowledge [13]. Additionally, spaced repetition is inherently beneficial, which could have improved performance results independently of teaching sessions [14]. Shuffling answer choices between assessments and releasing questions only at the conclusion of the learning cycles were strategies used to decrease the impact of repetition. Students also could have independently reviewed material between sessions, improving their performance; however, improvements were not reliable enough to attribute to this effect, and sessions were held in close proximity to reduce this bias. Small cohorts not only potentially limit the statistical analysis but also limit the psychological safety for learners to participate in thoughtful metacognitive discussions in CBL due to social pressures to seem competent. Larger cohorts would reduce the risk of error in the statistical analyses, confirming or emphasizing our findings. There are also limitations to the external validity of this data due to this particular PA program having a large percentage of nontraditional and veteran learners, limiting its generalizability. 

Further research is needed to explore long-term learning outcomes, clinical application of teaching methods, and specific areas of best application for CBL methods. Additionally, further research into other competencies gained from case-based teaching methods, such as increased communication and teamwork skills, could provide evidence of other benefits to this teaching method [3]. There are many natural experiments that transitioning to a CBL curriculum could provide the substrate for: clinical competency compared to didactic-only cohorts at clinical entry, time to achieve competency in structured evaluations, and so many more. These longer-term outcomes would further bolster and support the use of this instructional strategy in this learning context. There also remain significant gaps in research surrounding the impact on the educator in using CBL, which could lead to decreased educator burnout and increased enthusiasm in teaching within medical education [15]. Overall, our results demonstrate the efficacy of adding case-based teaching alongside didactic teaching in pharmacology education for PA students based on both objective and subjective data. While learners often prefer didactic teaching due to its familiarity, clarity, and perceived usefulness in knowledge acquisition, CBL fosters higher-order thinking, clinical application, and the development of skills necessary for clinical practice, such as communication, decision-making in uncertainty, and teamwork. Such skills are difficult to assess with objective evaluations, but are paramount for the delivery of safe and effective medical care for future healthcare providers. 

## Conclusions

This study investigated the effect of CBL methods used in addition to traditional didactic-based teaching methods via multiple-choice question assessments as well as subjective participant survey responses. Our findings indicate that, overall, multiple-choice assessment scores increased with statistical significance over all six topics taught during the study; however, some topics showed more or less of an increase in multiple-choice scores after supplementary CBL. Our findings also suggest that most students believed the CBL sessions benefited their learning with regard to concept application and preparation for board examinations. This study addresses a gap in current literature through its use in PA education, as well as further clarifying the relationship between CBL methods in addition to, rather than in place of, didactic teaching, thus leading to greater learning outcomes.
